# Gene transfer of MRCKα rescues lipopolysaccharide-induced acute lung injury by restoring alveolar capillary barrier function

**DOI:** 10.1038/s41598-021-99897-3

**Published:** 2021-10-21

**Authors:** Jing Liu, David A. Dean

**Affiliations:** 1 Department of Pediatrics, School of Medicine and Dentistry, University of Rochester, 601 Elmwood Avenue, Box 850, Rochester, NY 14642 USA; 2 Department of Pharmacology and Physiology, School of Medicine and Dentistry, University of Rochester, Rochester, NY 14642 USA

**Keywords:** Gene therapy, Respiration, Respiratory distress syndrome

## Abstract

Acute Lung Injury/Acute Respiratory Distress Syndrome (ALI/ARDS) is characterized by alveolar edema accumulation with reduced alveolar fluid clearance (AFC), alveolar-capillary barrier disruption, and substantial inflammation, all leading to acute respiratory failure. Enhancing AFC has long been considered one of the primary therapeutic goals in gene therapy treatments for ARDS. We previously showed that electroporation-mediated gene delivery of the Na^+^, K^+^-ATPase β1 subunit not only increased AFC, but also restored alveolar barrier function through upregulation of tight junction proteins, leading to treatment of LPS-induced ALI in mice. We identified MRCKα as an interaction partner of β1 which mediates this upregulation in cultured alveolar epithelial cells. In this study, we investigate whether electroporation-mediated gene transfer of MRCKα to the lungs can attenuate LPS-induced acute lung injury in vivo. Compared to mice that received a non-expressing plasmid, those receiving the MRCKα plasmid showed attenuated LPS-increased pulmonary edema and lung leakage, restored tight junction protein expression, and improved overall outcomes. Interestingly, gene transfer of MRCKα did not alter AFC rates. Studies using both cultured microvascular endothelial cells and mice suggest that β1 and MRCKα upregulate junctional complexes in both alveolar epithelial and capillary endothelial cells, and that one or both barriers may be positively affected by our approach. Our data support a model of treatment for ALI/ARDS in which improvement of alveolar-capillary barrier function alone may be of more benefit than improvement of alveolar fluid clearance.

## Introduction

Acute Respiratory Distress Syndrome (ARDS) is a devastating clinical condition of acute respiratory failure^1,2^. It is characterized by pulmonary edema of noncardiogenic origin and a pathologic diffuse alveolar damage phenotype, primarily caused by alveolar capillary barrier dysfunction and protein rich fluid flooding into alveoli and lung interstitial space^1,2^. It is estimated that there are 190,000 cases of ARDS annually in the United States with a hospital mortality of up to 40%^3^, and even one cross-country study, LUNG SAFE, has reported a mortality as high as 46% for severe ARDS^4^. Since ARDS is a syndrome caused by significant environmental insults rather than a single gene mutation or mechanism, there is still no effective pharmacological therapy developed for ARDS and the clinical strategy mainly relies on supportive care and ventilatory management. In the evolving COVID-19 pandemic, a study reported 67% of critically ill patients with SARS-CoV2 pneumonia developed ARDS^5^, which further highlights the importance of developing novel treatments or therapies for ARDS.

In its early stage, ARDS is characterized by the significant influx of protein-rich edema fluid into alveolar spaces, which is followed by extensive release of inflammatory cytokines and neutrophil sequestration in the lung^1^. The abnormal edema accumulation in ARDS is not only due to impaired alveolar fluid clearance (AFC), but also due to alveolar capillary barrier disruption which further contributes to the edema fluid influx^1,6,7^. Therefore, effective AFC, repair of a functional alveolar-capillary barrier, and the decreased inflammatory response are considered primary therapeutic targets for ARDS.

In most patients with ARDS, AFC is impaired^8,9^. Thus, one main therapeutic approach has been to rescue or increase AFC through increasing the overall vectoral out flow of Na^+^ across the alveolar epithelium, driving edema fluid reabsorption following the induced osmotic gradient^10^. The Na^+^, K^+^-ATPase is the predominant factor controlling AFC since it is the major active Na^+^ transporter expressed in the epithelial basolateral membrane and is responsible for homeostasis^11^. However, its membrane abundance and activity is downregulated under the injury^12^. Experimental data indicates that directly overexpressing or indirectly upregulating activities of the Na^+^, K^+^-ATPase and other ion channels (e.g., epithelial sodium channel (ENaC), cystic fibrosis transmembrane regulator (CFTR)) significantly increases the active Na^+^ transport and thus, AFC in multiple animal models^13–15^. Our lab has shown that transthoracic electroporation can efficiently transfer genes, including the Na^+^, K^+^-ATPase, into animal lungs without inducing injury^16^. Gene transfer of the β1 subunit of the Na^+^, K^+^-ATPase (β1-Na^+^, K^+^-ATPase) not only increases fluid clearance in healthy rat lungs^16^, but also protected mice from subsequent lipopolysaccharide (LPS) induced injury and even treated previously existing LPS-induced lung injury in mice by enhancing AFC^17–19^. Moreover, this approach also was able to treat pigs with sepsis-ischemia/reperfusion injury-induced ARDS^19^.

Apart from impaired AFC, alveolar-capillary barrier dysfunction and its associated hyperpermeability contribute to pulmonary edema accumulation in the interstitial and alveolar space^1,6,9,20^. The expression and function of the Na^+^, K^+^-ATPase is not only critical to AFC, but is also closely involved in the regulation of epithelial barrier integrity^12,21,22^. We previously demonstrated that when the β1-Na^+^, K^+^-ATPase was overexpressed by electroporation before or after induction of lung injury by LPS, tight junction (ZO-1, occludin) protein expression/complex formation and pulmonary barrier function increased, as demonstrated by decreased lung permeability, edema accumulation, total protein and cellularity in bronchoalveolar lavage (BAL) fluid, and improved overall outcome of lung injury^18^. To characterize the mechanism by which the β1-Na^+^, K^+^-ATPase increased tight junction and barrier function, we identified CDC42 binding protein kinase alpha (MRCKα) as an interacting partner of the β1-Na^+^, K^+^-ATPase through mass spectrometry^23^. MRCKα is a downstream effector of cdc42, a Rho family small GTPase which regulates cytoskeletal organization in a number of cell physiological processes, including polarity^24^, migration^25^, and junction formation^26,27^. MRCKα-mediated activation of non-muscle myosin II (NM-II) at cell–cell contacts promotes the reorganization of the actin cytoskeleton which is responsible for both endothelial and epithelial barrier formation and regulation^28,29^. Silencing MRCKα by siRNA or blocking its activity with a pharmacological inhibitor abrogated β1 increased tight junction protein expression and barrier integrity in the cultured alveolar type I (ATI) cells, suggesting the Na^+^, K^+^-ATPase β1 subunit acts through MRCKα to increase tight junction and barrier function. Overexpression of MRCKα alone into cultured ATI cells significantly increased localization of ZO-1 to cell–cell contacts and increased the basal transepithelial electrical resistance (TEER), indicating that overexpression of MRCKα alone is sufficient to induce tight junction formation and increase epithelial barrier integrity. Histologically, immunofluorescence stained lung tissues from patients with ARDS showed decreased expression of MRCKα compared to healthy controls^23^, further suggesting a role in ARDS pathophysiology.

In this study, we investigated whether electroporation mediated gene transfer of MRCKα could attenuate LPS-induced acute lung injury in vivo. Further, we tested whether overexpressing MRCKα in combination with β1-Na^+^, K^+^-ATPase could provide even greater treatment benefits for pre-existing LPS induced lung injury compared to either β1-Na^+^, K^+^-ATPase or MRCKα alone. Our results demonstrated that overexpression of MRCKα alone was as effective as overexpressing β1-Na^+^, K^+^-ATPase alone or in combination with MRCKα in attenuating LPS-increased pulmonary edema (Wet/Dry ratio), lung permeability/leakage, total protein concentration and cellularity of BAL fluid, and improving overall outcome of lung injury. In addition, we found that gene transfer of MRCKα did not enhance AFC, indicating its role in regulating alveolar capillary barrier integrity without promoting ion transport.

## Results

### Overexpression of MRCKα increases tight junction protein expression in healthy mouse lungs

**Figure 1:**
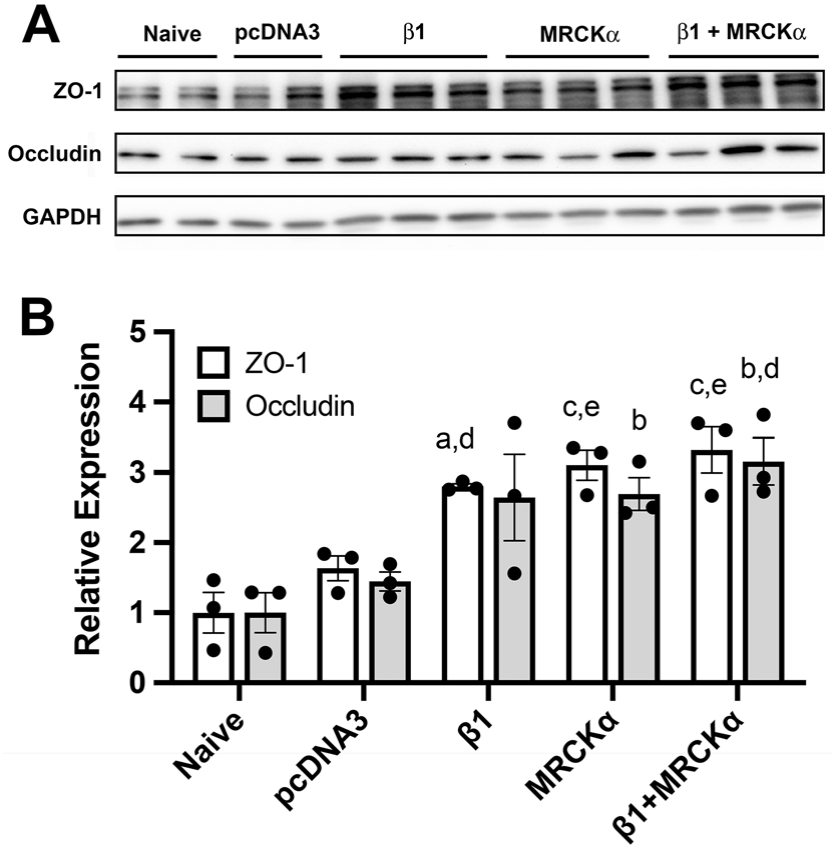
Overexpression MRCKα increases tight junction protein expression in healthy mouse lungs. Plasmids (100 µg each) expressing either no insert (pcDNA3), the β1 subunit of the Na^+^,K^+^-ATPase (β1), MRCKα, or β1 and MRCKα were delivered in 50 µl to the lungs of C75B6 mice (n = 3) by aspiration followed immediately by electroporation (8 pulses of 10 ms duration each and 200 V/cm). Two days later, lungs were perfused with PBS and lysates were prepared for analysis by Western Blot (**A**). Levels of expression were normalized to GAPDH as a loading control and the relative expression of ZO-1 (white bars) and Occludin (grey bars) are shown as mean ± SEM (**B**). All experiments were carried out three times and a representative experiment is shown. One-way ANOVA with post-hoc Tukey’s multiple comparisons was used for statistical analysis; a, p < 0.01 compared to naïve; b, p < 0.05 compared to naïve; c, p < 0.001 compared to naïve; d, p < 0.05 compared to pcDNA3; e, p < 0.01 compared to pcDNA3.

We previously have reported that the induction of tight junction proteins and their membrane localization by the Na^+^, K^+^-ATPase β1 subunit in cultured alveolar epithelial cells is mediated through the kinase MRCKα and further that forced expression of MRCKα in these cells was sufficient to increase tight junction protein levels^23^. To determine whether MRCKα overexpression also leads to increased tight junction protein levels in the healthy mouse lung, we delivered MRCKα-expressing plasmids to the lung by transthoracic electroporation and evaluated the relative expression of ZO-1 and occludin, two tight junction proteins 2 days later (Fig. 1). As we have shown previously in cells and mouse lungs, similar overexpression of the β1-Na^+^, K^+^-ATPase increased the levels of ZO-1 and occludin one to twofold (2.8 ± 0.04, p < 0.01, and 2.64 ± 0.64, p = 0.056, respectively), compared to naive, whereas gene transfer of a non-expressing empty plasmid (pcDNA3) had no statistically significant effect on the levels of either protein^18^. Overexpression of MRCKα increased ZO-1 and occludin expression relative to naïve similarly to that seen with β1 (3.1 ± 0.21, p < 0.001, and 2.69 ± 0.23, p < 0.05, respectively). To ask whether overexpression of both β1-Na^+^, K^+^-ATPase and MRCKα could lead to expression that was greater than either alone, both plasmids were delivered to mice (both plasmids delivered at the same levels as for individual delivery), but lead to similar overexpression as for either protein individually (3.32 ± 0.33, p < 0.001, and 3.16 ± 0.34, p < 0.05, respectively, compared to naïve). These results show that increased level of ZO-1 and occludin following overexpression of MRCKα alone was comparable to that caused by overexpression of β1-Na^+^, K^+^-ATPase alone or in combination with MRCKα, and are consistent with the engagement of β1-Na^+^, K^+^-ATPase/MRCKα axis observed in cultured alveolar epithelial cells to enhance tight junctions^23^.

### Overexpression of MRCKα alone or in combination with β1-Na^+^, K^+^-ATPase restores ZO-1 and occludin expression in previously injured mouse lungs

**Figure 2:**
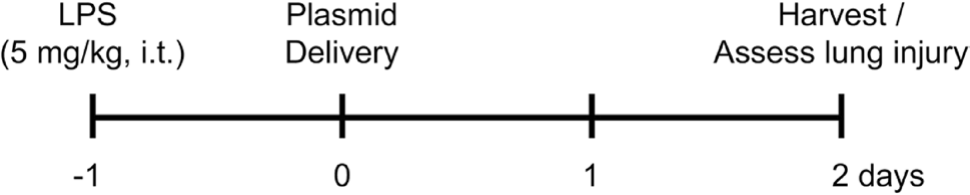
Experimental timeline for treatment of lung injury. Lung injury was established in mice by 5 mg/kg LPS administered by aspiration and 1 day later, plasmids (either β1, MRCKα, or a combination of the two; 100 µg in 50 µl PBS) were aspirated into the lungs and electroporated using electrodes placed on either side of the chest. Two days after gene delivery, lung injury was assessed.

**Figure 3:**
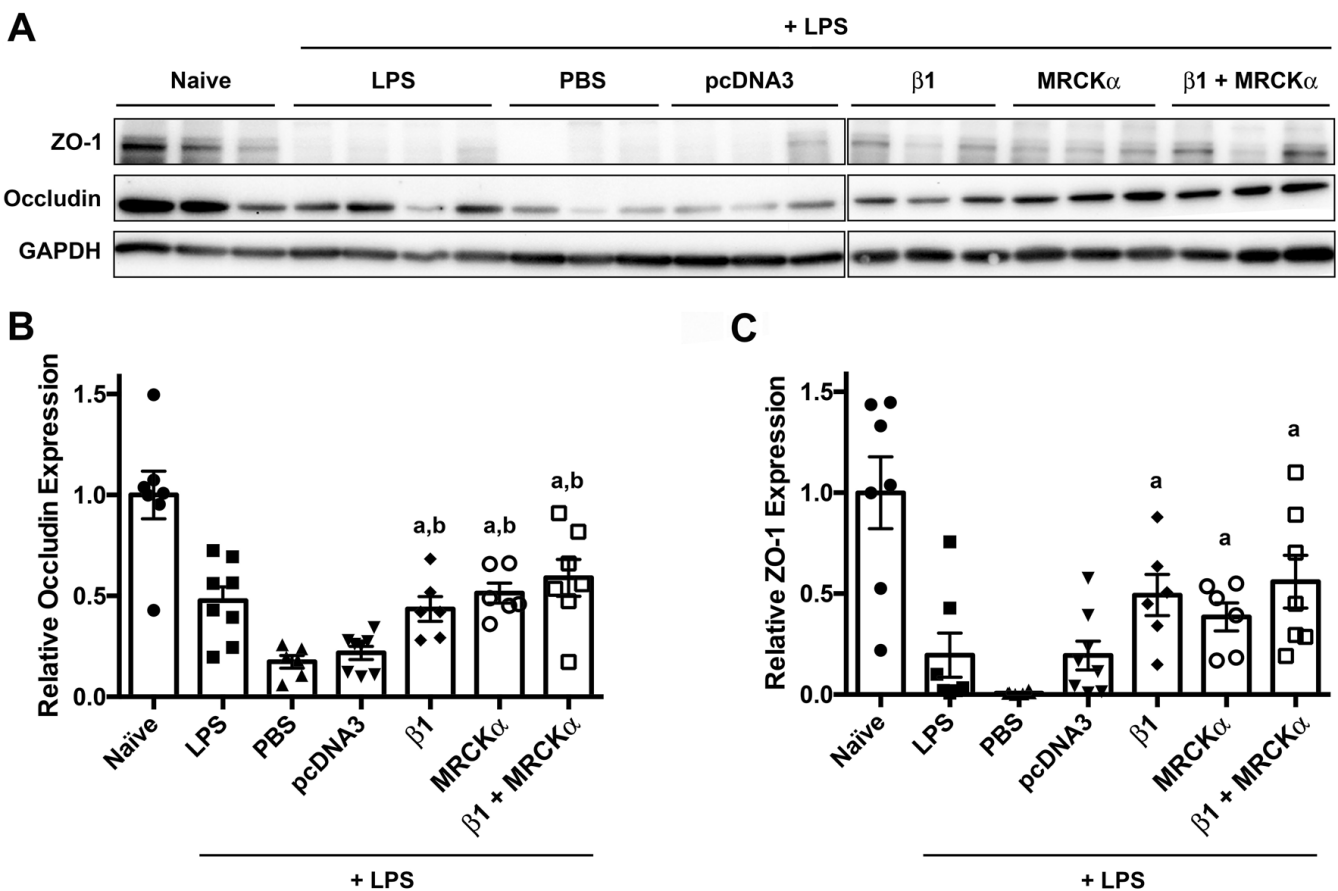
Overexpression of MRCKα restores ZO-1 and occludin expression in previously injured mouse lungs. Lung injury was established in C57B6 mice (n = 6–8) by aspiration of LPS (5 mg/kg) and 1 day later plasmids (100 µg each) expressing either no insert (pcDNA3), the β1 subunit of the Na^+^,K^+^-ATPase (β1), MRCKα, or β1 and MRCKα were delivered in 50 µl to the lungs by aspiration followed immediately by electroporation (8 pulses of 10 ms duration each and 200 V/cm). Two days later (3 days after LPS administration), lungs were perfused with PBS and lysates were prepared for analysis by Western Blot (**A**). Levels of expression were normalized to GAPDH as a loading control and the relative expression of Occludin (**B**) and ZO-1 (**C**) are shown as mean ± SEM. All experiments were carried out three times and representative experiments are shown. One-way ANOVA with post-hoc Tukey’s multiple comparisons was used for statistical analysis; a, p < 0.05 compared to naïve; b, p < 0.05 compared to pcDNA3.

We have shown that electroporation-mediated gene transfer of the β1-Na^+^, K^+^-ATPase can treat pre-established LPS-induced lung injury in mice^18^. In LPS injured mouse lungs, tight junction expression levels are decreased, as is barrier function, but upon gene delivery of the β1-Na^+^, K^+^-ATPase to LPS-injured lungs, tight protein levels were partially restored^18^. Since the increased levels of tight junction proteins (ZO-1 and occludin) following gene delivery of the β1-Na^+^, K^+^-ATPase is mediated through MRCKα in cells^23^ and in healthy lungs (Fig. 1), we next asked whether overexpression of MRCKα could similarly rescue the decreased level of ZO-1 and occludin in LPS injured mouse lungs. Lung injury was induced by intratracheal administration of LPS (5 mg/kg body weight) and 1 day later when edema, infiltrating inflammatory cells, and injury is present^17^, plasmids expressing the β1-Na^+^, K^+^-ATPase, MRCKα, or a mixture of the two were delivered by aspiration and electroporation (Fig. 2). Forty-eight hours later, the lungs were harvested for western blots (Fig. 3). As seen previously, levels of ZO-1 and occludin are both decreased in animals with LPS-induced lung injury at 3 days compared to naïve animals. Treatment of LPS-injured mice with either PBS alone (no electroporation) or with an empty plasmid (pcDNA3) had no beneficial effects on the levels of either protein, nor were they statistically more reduced compared to LPS alone. By contrast, electroporation-mediated delivery of MRCKα increased expression of both proteins by twofold compared to PBS or empty plasmid (p < 0.05 for ZO-1 or occludin compared to PBS or pcDNA3). Similarly, overexpression of β1-Na^+^, K^+^-ATPase gave a similar increase in the levels of both proteins compared to PBS or pcDNA3 (p < 0.05), as did the combined delivery and expression of both β1-Na^+^, K^+^-ATPase and MRCKα. These results indicate that MRCKα overexpression can increase levels of tight junction proteins in animals with existing lung injury. Further, since co-expression of both β1 and MRCKα did not lead to increases in ZO-1 or occludin expression beyond those seen with either gene alone, this would suggest that the increased expression seen of both proteins may be maximal in the context of the injured lung.

### Electroporation-mediated gene transfer of MRCKα attenuates the increased lung permeability in LPS-injured mice

**Figure 4:**
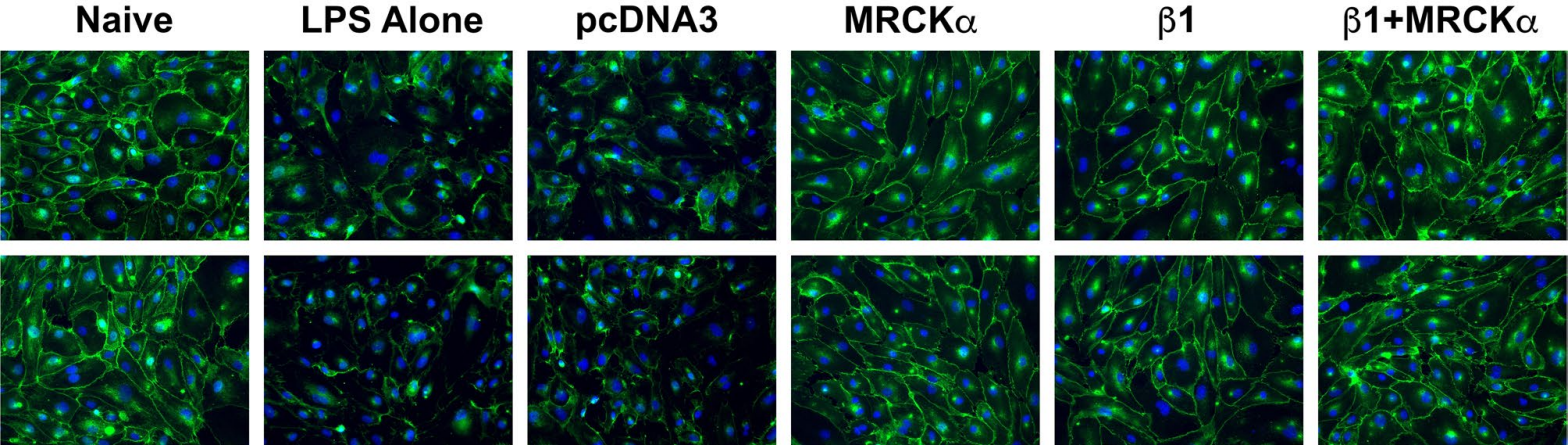
Overexpression of MRCKα or the Na^+^,K^+^-ATPase β1 subunit in microvascular endothelial cells attenuates LPS-induced reduction of VE-cadherin expression. Human MVECs grown on coverslips were transfected with plasmids (2 µg/well) expressing either no insert (pcDNA3), MRCKα, the β1 subunit of the Na^+^,K^+^-ATPase (β1), or β1 and MRCKα. Forty-eight hours later, cells were treated with nothing (Naïve) or LPS (1 µg/ml) for 5 h prior to fixation and immunofluorescent staining for VE-cadherin. The experiment was carried out on triplicate coverslips in three different experiments and representative images from two different wells are shown.

**Figure 5:**
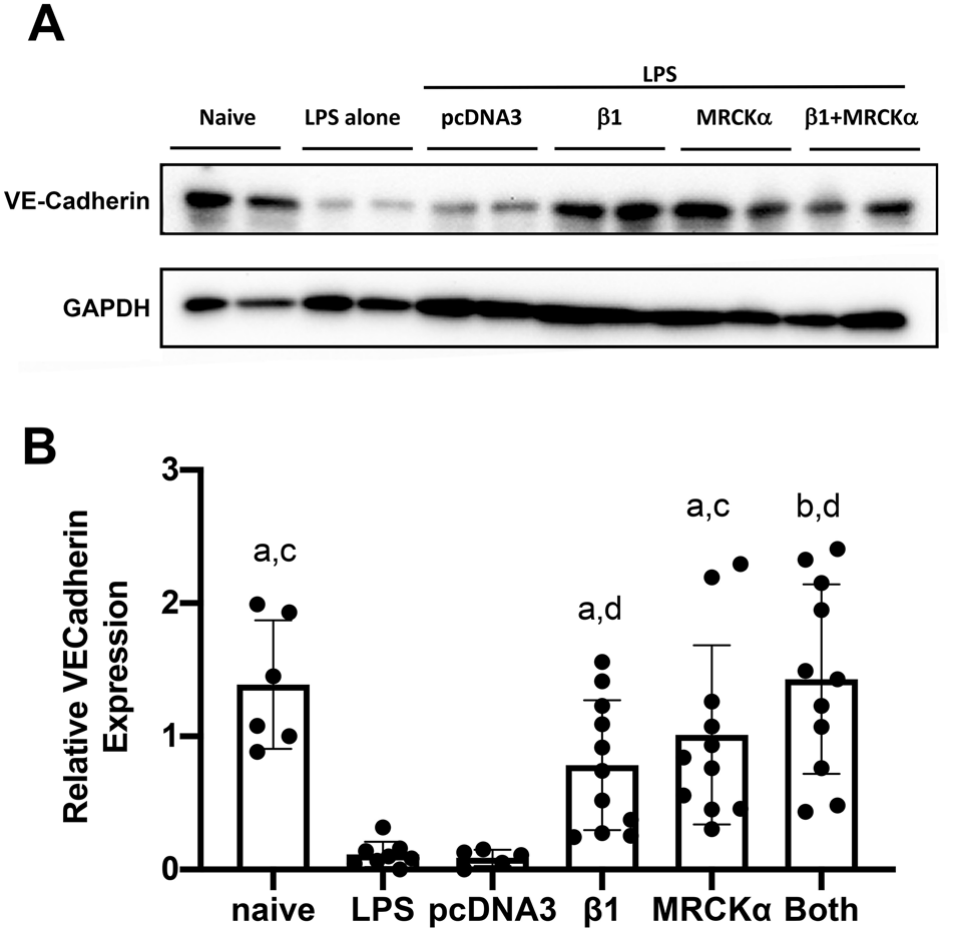
Electroporation-mediated gene transfer of MRCKα to mice with existing lung injury attenuates LPS-induced reduction in VE-cadherin. Lung injury was established in C57B6 mice (n = 6–12) by aspiration of LPS (5 mg/kg) and 1 day later plasmids (100 µg each) expressing either no insert (pcDNA3), the β1 subunit of the Na^+^,K^+^-ATPase (β1), MRCKα, or β1 and MRCKα were delivered in 50 µl to the lungs by aspiration followed immediately by electroporation (8 pulses of 10 ms duration each and 200 V/cm). Two days later (3 days after LPS administration), lungs were perfused with PBS and lysates were used for Western blots of VE-cadherin expression (**A**). Levels of expression were normalized to GAPDH as a loading control and the relative expression of VE-cadherin (**B**) is shown as mean ± SEM. All experiments were carried out three times and representative experiments are shown. One-way ANOVA with post-hoc Tukey’s multiple comparisons was used for statistical analysis; a, p < 0.05 compared to LPS only; b, p < 0.01 compared to LPS only; c, p < 0.05 compared to pcDNA3; and d, p < 0.01 compared to pcDNA3.

Our previously published data showed that overexpression of the β1-Na^+^, K^+^-ATPase attenuated the increased alveolar-capillary permeability seen in LPS-injured lungs, consistent with the ability of the β1 subunit to upregulate tight junction protein expression in mouse lungs with existing lung injury^18^. We also have shown in cultured AT1 cells that silencing of MRCKα by siRNA abrogates β1 increased alveolar epithelial barrier integrity (measured by TEER) and upregulation of tight junction proteins by β1^23^. Further, gene transfer of MRCKα alone has been shown to be sufficient to increase the epithelial barrier integrity and tight junction complex formation in cultured ATI cells^23^. These data suggest that MRCKα mediates the upregulation of tight junction proteins and barrier function either by its overexpression or by that of the β1 subunit, at least in vitro. While we also have shown that overexpression of β1 in cultured human pulmonary artery endothelial cells increases transcription and protein levels of ZO-1 and occludin, we have not evaluated whether gene transfer in vitro can protect endothelial cells from LPS-induced barrier disruption^23^. Cultured mouse microvascular endothelial cells were transfected with plasmids expressing MRCKα and/or β1 and 48 h later challenged with LPS (1 µg/ml) for 5 h, at which point cells were fixed and stained for expression of VE-cadherin (Fig. 4). In naïve cells, VE-cadherin staining at the cell membrane is clear and is greatly decreased upon stimulation with LPS. However, when either MRCKα, β1, or a combination of the two genes was overexpressed in these cells, the LPS-induced decrease in VE-cadherin was largely attenuated, suggesting that both genes can regulate junctional complexes in endothelial cells as well as epithelial cells^23^. Based on this, we evaluated whether MRCKα and/or β1 gene transfer to LPS pre-injured mouse lungs also resulted in endothelial delivery and restored the decreased junctional complexes in vivo. To this end, we quantified levels of VE-cadherin, a protein that has been shown to be a good indicator of endothelial cell barrier dysfunction, in mice that were injured with intratracheal LPS and 24 h later subjected to electroporation-mediated gene transfer. When lung homogenates were probed for VE-cadherin by Western blot, we found that gene transfer of either MRCKα, β1, or a mixture of MRCKα and β1, but not an empty plasmid (pcDNA3) reversed the LPS-induced reductions in VE-cadherin seen in mouse lungs (Fig. 5). This indicates that electroporation-mediated gene transfer can deliver genes to multiple cell layers in the lung and that MRCKα and β1 can rescue the decreased levels of endothelial junctional complexes.

**Figure 6:**
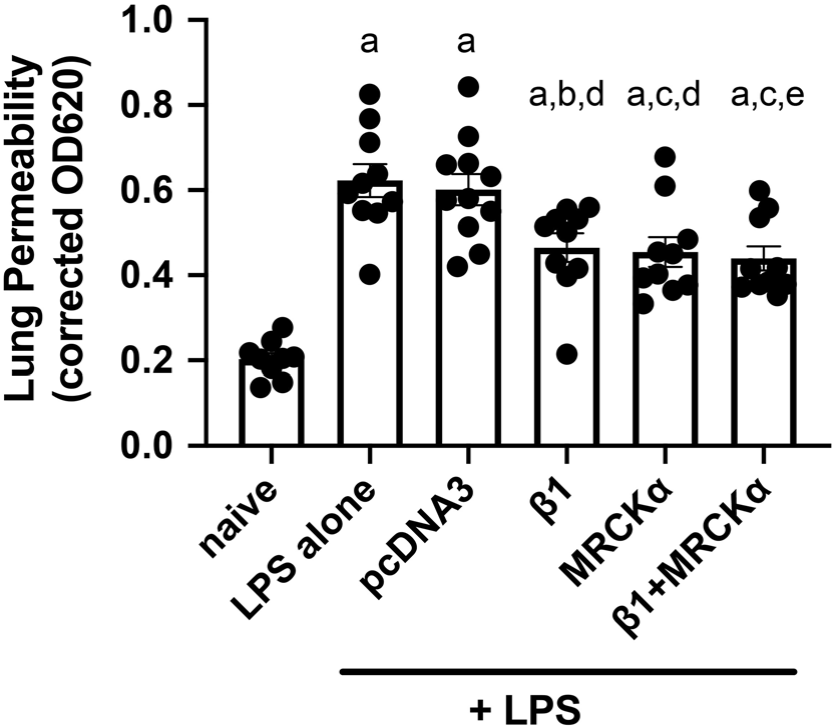
Electroporation mediated gene transfer of MRCKα attenuates lung leakage in lungs of mice previously injured with LPS. Lung injury was established in C57B6 mice (n = 9–11) by aspiration of LPS (5 mg/kg) and 1 day later plasmids (100 µg each) expressing either no insert (pcDNA3), the β1 subunit of the Na^+^, K^+^-ATPase (β1), MRCKα, or β1 and MRCKα were delivered in 50 µl to the lungs by aspiration followed immediately by electroporation (8 pulses of 10 ms duration each and 200 V/cm). Forty-seven hours later, Evans Blue Dye (30 mg/kg) was administered by tail vein injection and one hour later, lungs were perfused with PBS and harvested for Evans Blue Dye extraction. Lung permeability was evaluated by quantifying the absorbance of extracted Evans Blue Dye and shown as mean ± SEM. All experiments were carried out three times and a representative experiment is shown. One-way ANOVA with post-hoc Tukey’s multiple comparisons was used for statistical analysis; a, p ≤ 0.0001 compared to naïve; b, p < 0.05 compared to LPS; c, p < 0.01 compared to LPS; d, p < 0.05 compared to pcDNA3; e, p < 0.01 compared to pcDNA3.

To further determine whether overexpression of MRCKα could attenuate the increased pulmonary barrier leakage in pre-injured living animals, lung permeability was measured by the leakage of EBD labeled albumin from blood into airways^30^. As in Fig. 2, mouse lungs were injured with LPS and 1 day later electroporated with MRCKα, β1, or control plasmids and 47 h later evaluated for extravascular EBD accumulation following tail-vein injection of EBD (Fig. 6). Compared to the naïve group (0.203 ± 0.015), LPS induced threefold more leakage of EBD into the lung (0.623 ± 0.039), indicating alveolar capillary barrier disruption (p < 0.0001). Gene transfer of the empty vector pcDNA3 into lungs injured 24 h prior with LPS resulted in no change in lung permeability to EBD (0.601 ± 0.039) compared to LPS only. However, gene transfer of MRCKα significantly reduced the LPS-induced lung leakage to 0.455 ± 0.035, compared with LPS only (p < 0.01) or with pcDNA3 (p < 0.05). Electroporation-mediated gene delivery of the β1-Na^+^, K^+^-ATPase to LPS-injured lungs showed similar activity regarding permeability as did MRCKα (0.465 ± 0.033, p < 0.05 compared to LPS or pcDNA3). Co-administration of plasmids expressing β1 with MRCKα showed no further reduction in permeability compared to either plasmid alone (0.439 ± 0.028, p < 0.01 compared to LPS or pcDNA3). Collectively, these results indicate that gene transfer of MRCKα alone improves the alveolar capillary barrier function, pointing to the potential of MRCKα activation/overexpression to treat acute lung injury.

### MRCKα gene transfer to mouse lungs with existing LPS-induced injury can reduce pulmonary edema

**Figure 7:**
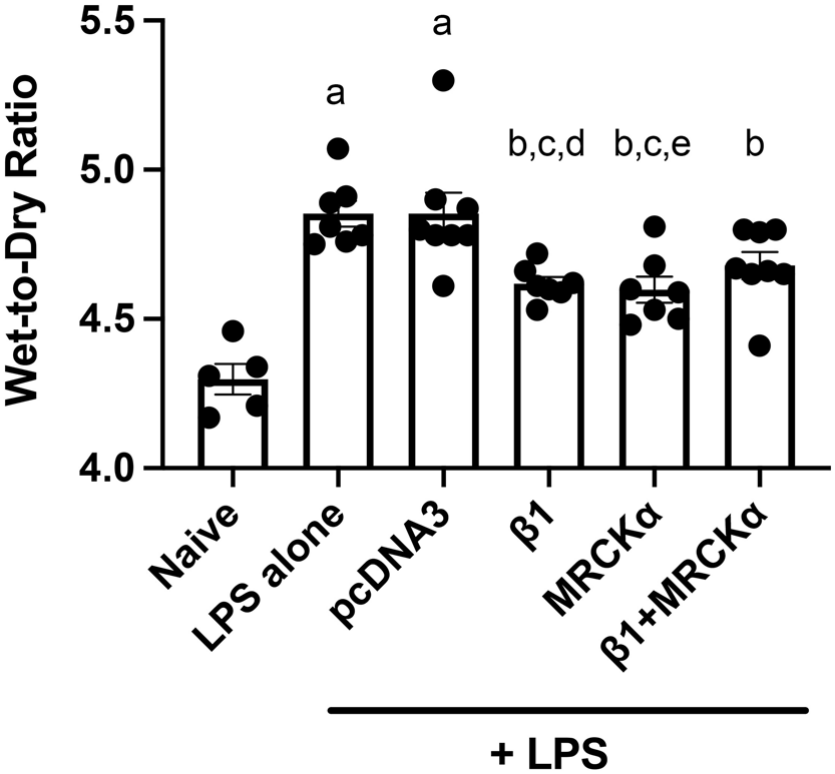
Electroporation mediated gene transfer of MRCKα attenuates lung edema fluid accumulation in previously injured lungs. Lung injury was established in C57B6 mice (n = 5–8) by aspiration of LPS (5 mg/kg) and 1 day later plasmids (100 µg each) expressing either no insert (pcDNA3), the β1 subunit of the Na^+^,K^+^-ATPase (β1), MRCKα, or β1 and MRCKα were delivered in 50 µl to the lungs by aspiration followed immediately by electroporation (8 pulses of 10 ms duration each and 200 V/cm). Two days later (3 days after LPS administration), wet to dry ratios were determined as a measure of pulmonary edema fluid and shown as mean ± SEM. All experiments were carried out three times and a representative experiment is shown. One-way ANOVA with post-hoc Tukey’s multiple comparisons was used for statistical analysis; a, p < 0.0001 compared to naïve; b, p < 0.01 compared to naïve; c, p < 0.05 compared to LPS; d, p < 0.05 compared to pcDNA3; e, p < 0.01 compared to pcDNA3.

We next determined whether MRCKα could ameliorate the overall lung edema accumulation following lung injury. As above, lung injury was induced by LPS, 1 day later plasmids were transferred to the mice, and lungs were harvested for gravimetric analysis and wet to dry ratios 2 days after gene transfer (Fig. 7). Compared with the naïve group (4.298 ± 0.051), mice injured with LPS showed an increased wet to dry ratio of 4.853 ± 0.043 (p < 0.0001), indicating a significant accumulation of edema fluid in the lung. Consistent with our previously published data, gene transfer of the empty vector plasmid pcDNA3 mice showed no change in the wet to dry ratio (4.853 ± 0.071) compared with LPS alone, whereas gene transfer of the MRCKα plasmid significantly decreased the wet to dry ratio to 4.599 ± 0.044 (p < 0.05 and p < 0.01 compared to LPS alone and LPS + pcDNA3, respectively). Similar to mice receiving the MRCKα plasmid, lungs electroporated with β1 subunit plasmid also showed significantly reduced pulmonary edema 4.619 ± 0.022 (p < 0.05 compared to LPS or pcDNA3). While gene transfer of MRCKα in combination with β1 plasmid reduced the wet to dry ratio (4.679 ± 0.046), this only trended to significance (p < 0.066 and p < 0.068, compared to LPS alone or LPS + pcDNA3, respectively). Thus, MRCKα overexpression attenuates edema accumulation in the injured lungs.

### Gene transfer of MRCKα attenuates inflammation in LPS injured lungs

**Figure 8:**
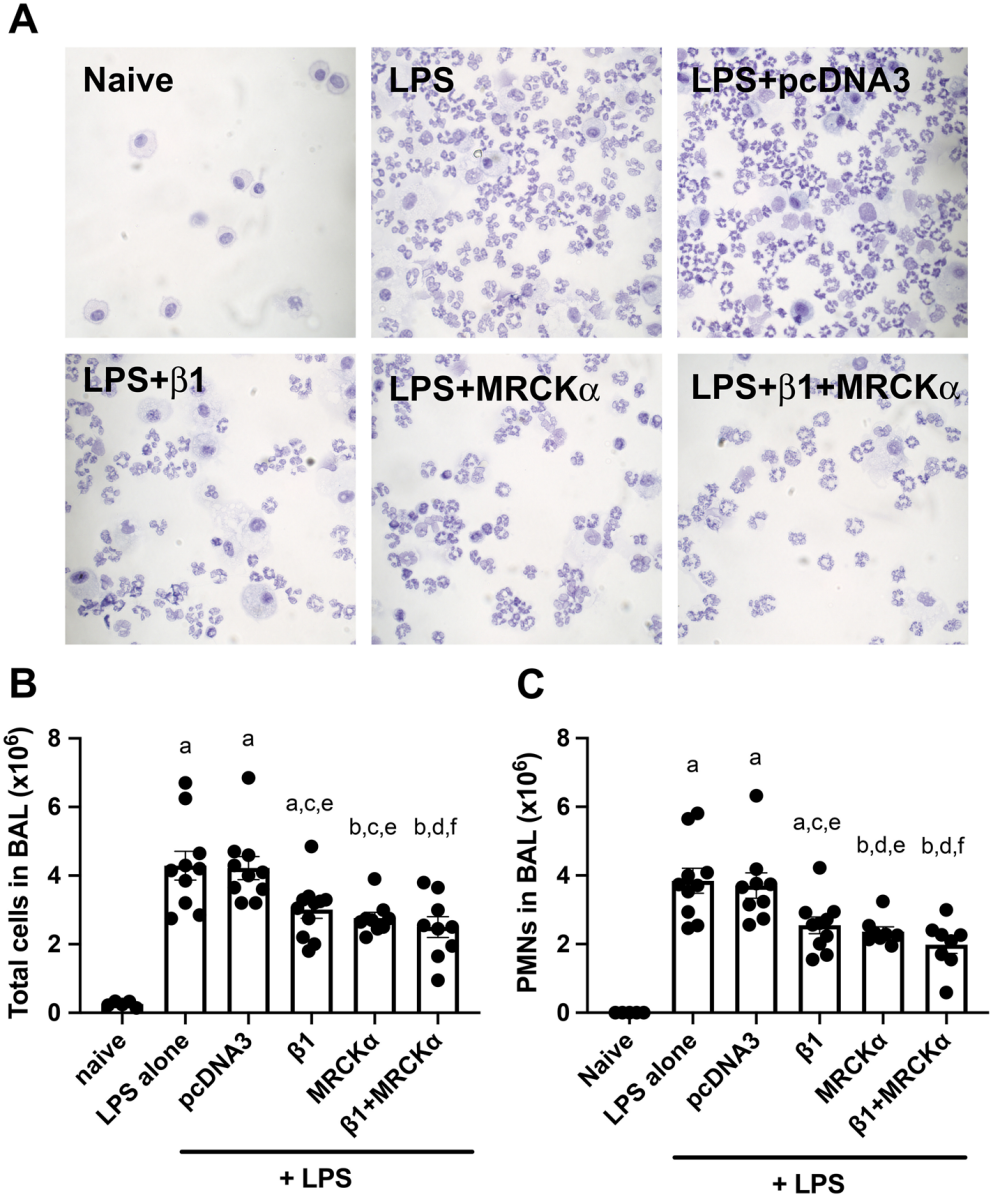
Gene delivery of MRCKα to mice with existing lung injury attenuates inflammatory cell infiltration. Lung injury was established in C57B6 mice (n = 8–10) by aspiration of LPS (5 mg/kg) and 1 day later plasmids (100 µg each) expressing either no insert (pcDNA3), the β1 subunit of the Na^+^,K^+^-ATPase (β1), MRCKα, or β1 and MRCKα were delivered in 50 µl to the lungs by aspiration followed immediately by electroporation (8 pulses of 10 ms duration each and 200 V/cm). Naïve mice (n = 5) received no LPS or DNA. Two days later (3 days after LPS administration), lungs were lavaged with PBS and BAL fluid was collected and analyzed for cellularity by cytospin followed by Diff-quik staining (**A**). All experiments were carried out three times and a representative experiment is shown. Total cells were quantified in the BAL fluid and shown as mean ± SEM (**B**). One-way ANOVA with post-hoc Tukey’s multiple comparisons was used for statistical analysis; a, p < 0.0001 compared to naïve; b, p < 0.001 compared to naïve; c, p < 0.05 compared to LPS; d, p < 0.01 compared to LPS; e, p < 0.05 compared to pcDNA3; f, p < 0.01 compared to pcDNA3. The number of PMNs in the BAL fluid were also quantified and shown as mean ± SEM (**C**). One-way ANOVA with post-hoc Tukey’s multiple comparisons was used for statistical analysis; a, p < 0.0001 compared to naïve; b, p < 0.01 compared to naïve; c, p < 0.05 compared to LPS; d, p < 0.01 compared to LPS; e, p < 0.05 compared to pcDNA3;f, p < 0.01 compared to pcDNA3.

**Figure 9:**
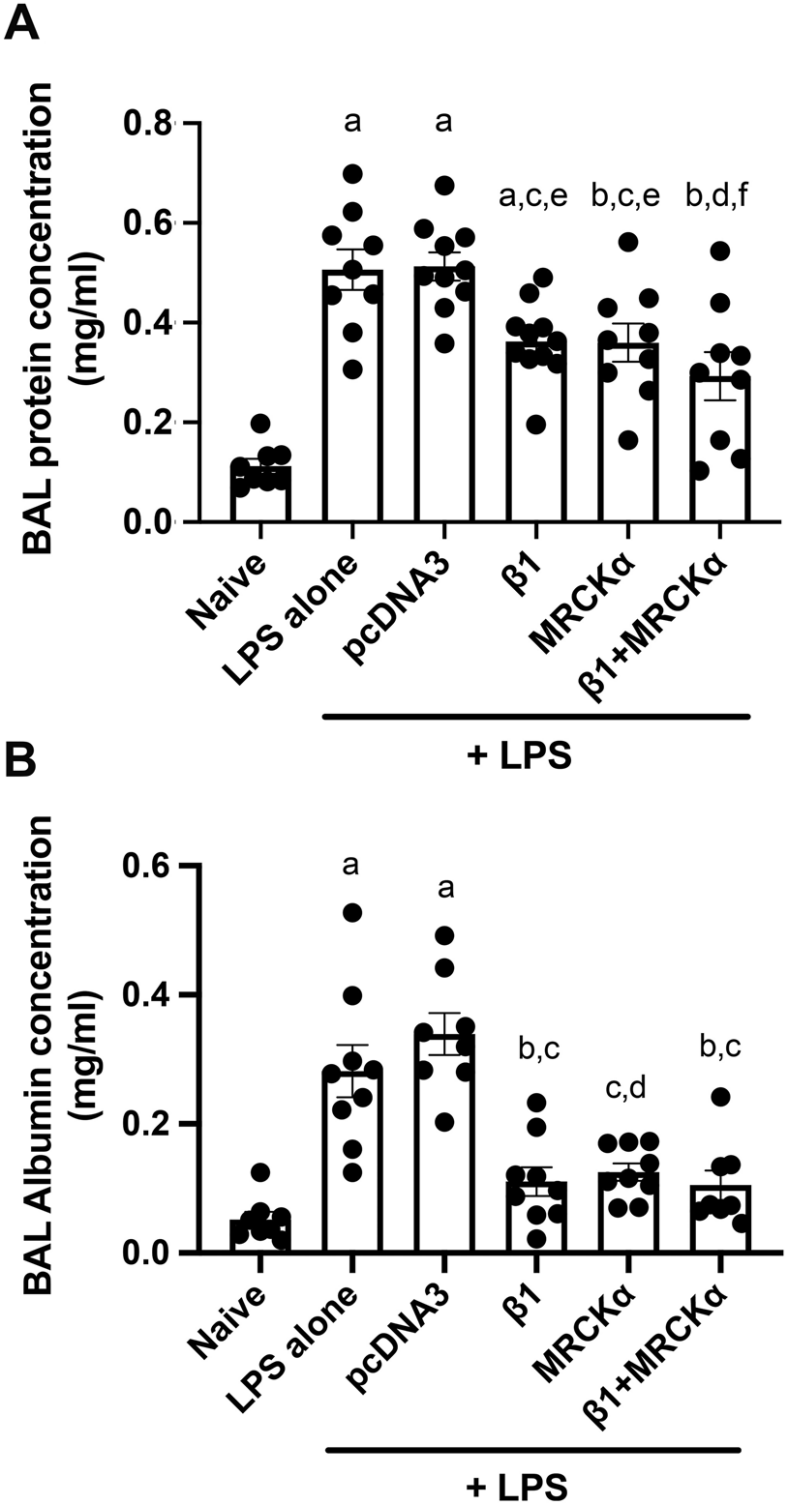
MRCKα gene transfer reduces BAL protein levels in previously injured mice. Lung injury was established in C57B6 mice (n = 8–9) by aspiration of LPS (5 mg/kg) and 1 day later plasmids (100 µg each) expressing either no insert (pcDNA3), the β1 subunit of the Na^+^,K^+^-ATPase (β1), MRCKα, or β1 and MRCKα were delivered in 50 µl to the lungs by aspiration followed immediately by electroporation (8 pulses of 10 ms duration each and 200 V/cm). Naïve mice (n = 5) received no LPS or DNA. Two days later (3 days after LPS administration), lungs were lavaged with PBS and BAL fluid was collected and analyzed for total protein content, shown as mean ± SEM (**A**). All experiments were carried out three times and a representative experiment is shown. One-way ANOVA with post-hoc Tukey’s multiple comparisons was used for statistical analysis; a, p < 0.0001 compared to naïve; b, p < 0.01 compared to naïve; c, p < 0.05 compared to LPS; d, p < 0.001 compared to LPS; e, p < 0.05 compared to pcDNA3; f, p < 0.001 compared to pcDNA3. The concentration of albumin in the BAL fluid was quantified by ELISA and shown as mean ± SEM (**B**). One-way ANOVA with post-hoc Tukey’s multiple comparisons was used for statistical analysis; a, p < 0.001 compared to naïve; b, p < 0.001 compared to LPS; c, p < 0.0001 compared to pcDNA3; d, p < 0.01 compared to LPS.

BAL fluid harvested from mouse lungs was used to analyze inflammation and injury by measuring cellularity and protein concentration as a measure of barrier dysfunction. As expected, instillation of LPS induced accumulation of infiltrating cells, most of which were PMNs, and increased the levels of serum protein in the BAL (Figs. 8 and 9). Compared with LPS injured mice which received empty vector pcDNA3, mice electroporated with plasmid encoding β1-Na^+^, K^+^-ATPase showed a significant reduction in the number of total cells and PMNs in BAL fluid (Fig. 8). Similarly, transfer of the β1 plasmid also reduced the concentration of total protein (p < 0.05) as well as that specifically of serum albumin in the BAL compared to LPS alone or LPS + pcDNA3 (p < 0.001 and p < 0.0001, respectively). More importantly, gene transfer of MRCKα alone or in combination with β1-Na^+^, K^+^-ATPase decreased the number of total cells in the BAL to 2.767 × 10^6^ ± 0.160 × 10^6^ and 2.500 × 10^6^ ± 0.305 × 10^6^, respectively, compared with mice with pcDNA3 delivery (4.220 × 10^6^ ± 0.336 × 10^6^; p < 0.05 and p < 0.01 for MRCKα or both plasmids) or LPS only (4.290 × 10^6^ ± 0.417 × 10^6^; p < 0.01 and p < 0.05 for MRCKα or both plasmids) (Fig. 8C). PMNs in the BAL were the predominant cell type and were markedly reduced to 2.365 × 10^6^ ± 0.139 × 10^6^ (MRCKα alone) or 1.983 × 10^6^ ± 0.253 × 10^6^ (MRCKα + β1- Na^+^, K^+^-ATPase), compared with 3.707 × 10^6^ ± 0.371 × 10^6^ of mice with control pcDNA3 (Fig. 5C). Total BAL protein and serum albumin were also significantly reduced in the lungs of mice that received either MRCKα alone or in combination with β1 plasmids (Fig. 9). However, gene transfer of both MRCKα and β1 plasmids failed to provide any significant benefit over either gene individually. Collectively, these results indicated that electroporation mediated gene transfer of MRCKα can treat injured lungs by reducing the numbers of infiltrating cells and decreasing extravasated serum protein in the BAL fluid.

### Levels of histologically evident lung injury in LPS injured lungs is reduced following gene transfer of MRCKα

**Figure 10:**
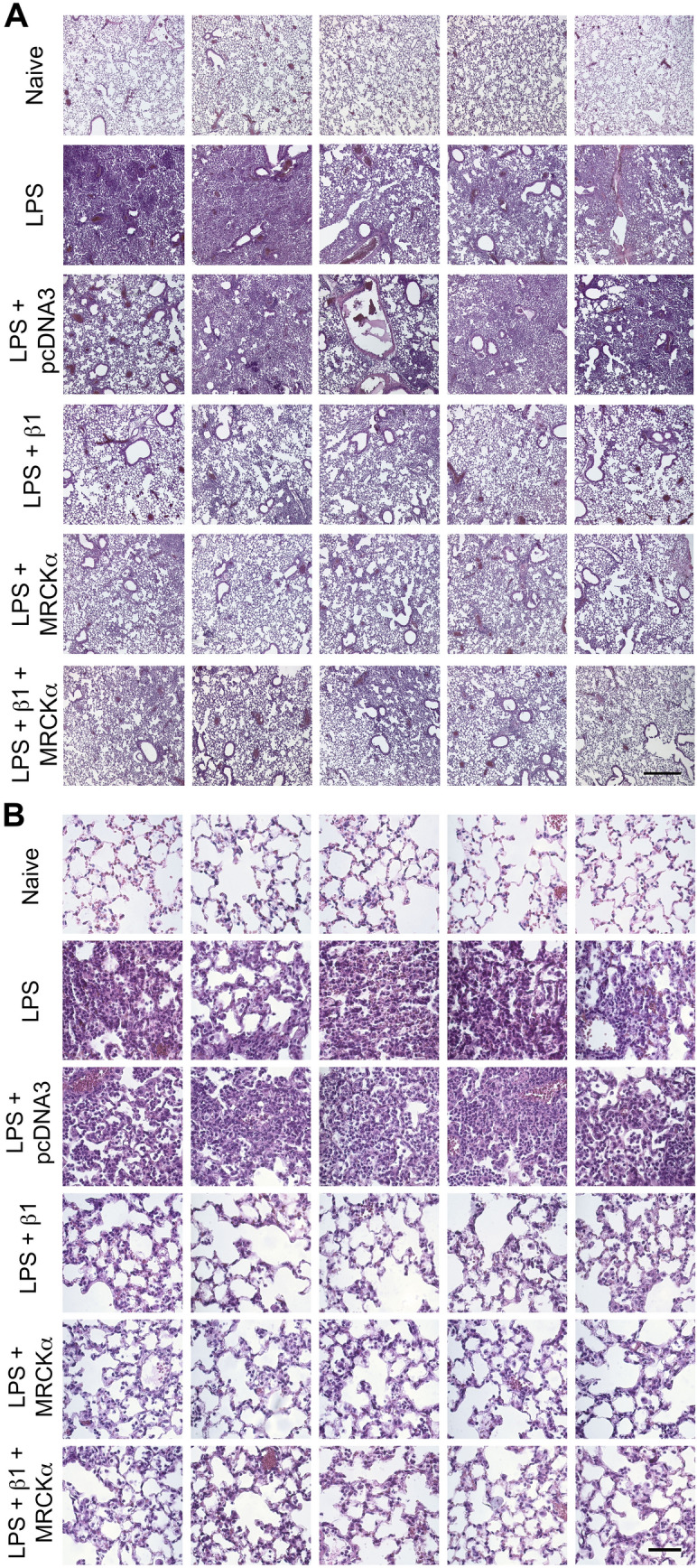
Electroporation-mediated gene transfer of MRCKα improves overall histology of mice with pre-existing LPS-induced lung injury. Lung injury was established in C57B6 mice (n = 6–8) by aspiration of LPS (5 mg/kg) and 1 day later plasmids (100 µg each) expressing either no insert (pcDNA3), the β1 subunit of the Na^+^,K^+^-ATPase (β1), MRCKα, or β1 and MRCKα were delivered in 50 µl to the lungs by aspiration followed immediately by electroporation (8 pulses of 10 ms duration each and 200 V/cm). Naïve mice (n = 7) received no LPS or DNA. Two days later (3 days after LPS administration), lungs were inflated to 20 cm H_2_O with 10% buffered formalin and processed for paraffin-embedding, sectioning, and hematoxylin and eosin staining. Sections from 5 representative animals are shown at ×50 (**A**) and ×400 (**B**) magnification. All experiments were carried out three times and a representative experiment is shown. Scale bar is 400 µm (**A**) and 50 µm (**B**).

To determine the overall injury in animals, we inflation fixed lungs and analyzed them histologically. Figure 8 shows random images taken of 5 different mice for each treatment cohort at low and high magnification. As expected, naïve mouse lungs show abundantly clear airspaces with undetectable infiltrating cells, thin alveolar walls, and no evidence of pulmonary edema or fibrin deposition in the alveoli, at either low or high magnification (Fig. 10). By contrast, when injured with LPS and no intervention, significant atelectasis, infiltrating cells (primarily PMNs), and thickened alveolar walls are clearly evident and relatively uniform throughout the lung, even at low magnification (Fig. 10A). Gene transfer of the empty plasmid pcDNA3 did not greatly affect any of these hallmarks of diffuse alveolar damage and ALI. However, animals receiving either MRCKα, β1, or a combination of the two, all showed greatly diminished injury in terms of reduced atelectasis, increased numbers of open and clear alveoli, and thin alveolar walls. While the lungs in all of these treated animals are not as pristine as those of naïve mice, they clearly show much less inflammation and damage than do the LPS-injured lungs of mice that received no therapeutic plasmids. Taken together with all the other measures of lung injury, these results demonstrate that degree of treatment of injured lungs following gene transfer of MRCKα is essentially comparable to that of lungs receiving the β1-Na^+^, K^+^-ATPase.

### Unlike the β1-Na^+^, K^+^-ATPase, MRCKα does not affect alveolar fluid clearance rates

**Figure 11:**
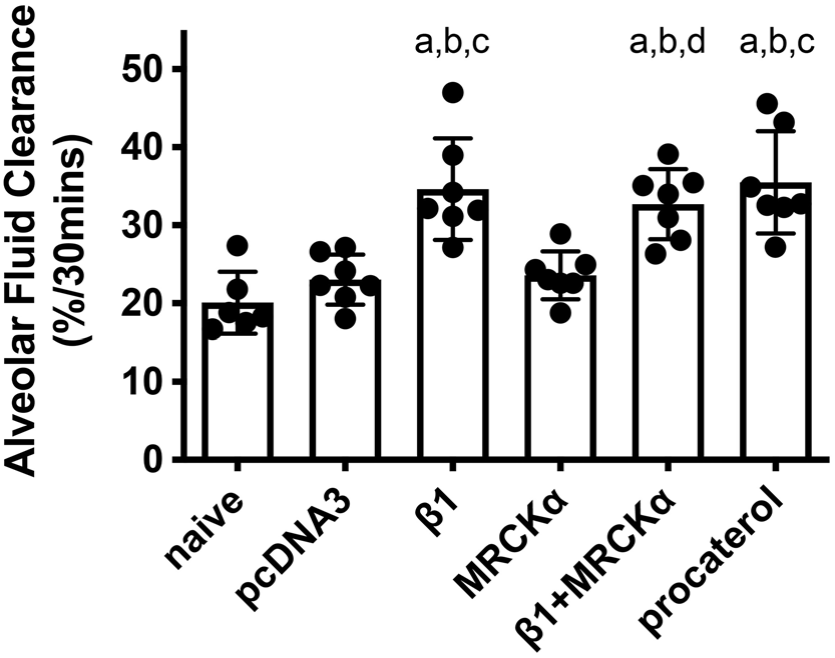
Electroporation mediated gene transfer of MRCKα has no effect on rates of alveolar fluid clearance. Plasmids (100 µg each) expressing either no insert (pcDNA3), the β1 subunit of the Na^+^,K^+^-ATPase (β1), MRCKα, or β1 and MRCKα were delivered in 50 µl to the lungs of C75B6 mice (n = 6–7) by aspiration followed immediately by electroporation (8 pulses of 10 ms duration each and 200 V/cm). Naïve mice received no DNA. Two days later, alveolar fluid clearance was measured in living mice and calculated based on the change in concentration of Evans Blue Dye-labeled albumin in an isosmolar (324 mOsm) instillate placed into the alveolar space and mechanically ventilated over a 30 min period. Procaterol (10^−8^ mol/L) was administered in the instillate and used as the positive control in a set of naïve mice. Rates of alveolar fluid clearance are shown as mean ± SEM. All experiments were carried out three times and a representative experiment is shown. One-way ANOVA with post-hoc Tukey’s multiple comparisons was used for statistical analysis; a, p < 0.01 compared to naïve; b, p < 0.001 compared to pcDNA3; c, p < 0.01 compared to MRCKα; d, p < 0.05 compared to MRCKα.

Historically, the leading therapeutic goal ARDS treatment has been to increase alveolar fluid clearance to promoting edema resolution. As the Na^+^, K^+^-ATPase is crucial for maintaining transepithelial osmotic pressure and continuously extrudes Na^+^, and hence, water, out of the alveoli and into the interstitium and capillaries, gene transfer of Na^+^, K^+^-ATPase subunits has been shown to significantly increase AFC in various experimental models^14–16,18,31,32^. To further investigate whether overexpression of MRCKα could accelerate fluid clearance in the lung, thereby accounting for a potential mechanism for the treatment effects of MRCKα, AFC was measured in living mice 2 days after gene transfer in healthy mice (Fig. 11).

Gene transfer of the β1 subunit significantly enhanced AFC (34.65 ± 2.46) by 73.5% and 51% compared with naïve (20.08 ± 1.64, p < 0.01) and mice that received empty plasmid pcDNA3 (20.04 ± 1.21, p < 0.001), respectively. There was no significant difference in AFC rates between naïve and pcDNA3 mice. Procaterol (10^−8^ mol/L), a specific β_2_-Adrenergic Receptor agonist, was used as a positive control and lead to a similar increase in AFC of 77.5% compared with naïve mice (p < 0.01). However, electroporation of MRCKα alone into mouse lungs failed to improve AFC (23.58 ± 1.16), compared with either naïve (p < 0.7859) or pcDNA3 (p < 0.9999) mice. By contrast, gene transfer of MRCKα in combination with β1 subunit plasmids increased AFC to 32.71 ± 1.70, comparable to that seen in mice treated with procaterol or those electroporated with the β1 plasmid alone. These results indicate that overexpression of MRCKα could not accelerate fluid clearance; the increased AFC in mice that received both MRCKα and β1 subunit plasmids was due to the enhanced ion transport activity caused by overexpression/activation of the Na^+^, K^+^-ATPase, independent of MRCKα.

## Discussion

In this study, we tested whether electroporation mediated gene transfer of MRCKα alone or in combination with β1-Na^+^, K^+^-ATPase could treat existing lung injury induced by intratracheally administered LPS. Consistent with the published data, gene transfer of β1-Na^+^, K^+^-ATPase reduced injury by improving AFC, restoring tight junction expression and pulmonary barrier function, and reducing inflammation in injured lungs. More importantly, overexpression of MRCKα alone attenuated prior LPS-induced injury to the same degree as the β1 subunit in terms of the levels of pulmonary edema, extravascular leakage of EBD labelled albumin, protein content and cellularity of BAL fluid, and histology, indicating that MRCKα could benefit the pre-injured lungs by reducing pulmonary edema, restoring lung barrier function and reducing inflammation. However, we found that simultaneous overexpression of both the β1-Na^+^, K^+^-ATPase and MRCKα gave no further enhancement of any outcome over that seen with overexpression of either protein alone, suggesting that gene transfer of either β1 or MRCKα alone might have reached the maximal therapeutic effect for treatment of ALI/ARDS. Moreover, based on the fact that overexpression of MRCKα had no effect on AFC, taken together, our results suggest that improvement in barrier function plays the predominant role in the treatment effect we see in injured lungs following either gene delivery of MRCKα or the β1 subunit of the Na^+^, K^+^-ATPase.

AFC has been long studied as the therapeutic target for ARDS due to the finding that the majority of patients with ARDS showed severe fluid clearance impairment^8,9^. However, the clinical benefits from upregulating AFC appear limited. β2 adrenergic agonists have been demonstrated to enhance AFC and transepithelial ion transport in cells and in mouse models through catecholamine-dependent activation of the cAMP pathway, leading to the upregulation of activity and membrane abundance of ENaC, chloride channels, and the Na^+^, K^+^-ATPase^11,33^. However, clinical trials of β2 adrenergic therapies failed to show significance in the primary outcomes, including ventilator-free days and mortality and contributed no benefit for patients with ARDS^34,35^. In the case of gene therapy, overexpression of the major apical epithelial sodium channel, ENaC, was able to greatly increase AFC in either transgenic mice or following gene transfer^18,36^. Further, Solnatide (AP301), a specific direct activator of ENaC, was able to restore alveolar epithelial barrier function, including occludin expression, and reduce pulmonary edema in a rat model of high altitude pulmonary edema, showing the therapeutic potential of ENaC regulation^37^. However, while gene transfer of ENaC could protect mouse lungs from subsequent LPS-induced injury by improving AFC, transfer of ENaC to lungs previously injured with LPS had absolutely no beneficial effect on lung injury, despite abundant overexpression of the protein, indicating that increased AFC is not sufficient to treat existing ALI, at least in this LPS injury model^18^. This failure of αENaC to treat LPS-injured lungs is somewhat surprising given the importance of ENaC in mediating vectoral Na^+^ transport and AFC, as evidenced by the fact that mice lacking αENaC die at birth due to alveolar flooding^38^. In contrast to αENaC, transfer of β1-Na^+^, K^+^-ATPase could not only protect mice from the following injury but also treat lungs with existing injury by rescuing lung barrier function as well as by improving AFC, suggesting that repair of the alveolar capillary barrier is critical for ALI/ARDS treatment.

Recent studies have shown that the β1-Na^+^, K^+^-ATPase upregulates tight junction abundance through MRCKα in cells and that overexpression of MRCKα itself is sufficient to improve barrier activity in vitro^23^. Further, in the present study we have shown that gene transfer of MRCKα into living lungs was unable to increase AFC compared with control mice despite abundant overexpression of MRCKα, yet was able to effectively treat the pre-injured lungs, as demonstrated as reduced lung edema accumulation and alveolar barrier leakage by restoring tight junction (ZO-1, occludin) protein expression. Taken together, these results suggest that restoring alveolar capillary barrier function, rather than enhancing alveolar fluid clearance, might be more important for ARDS treatment and account for the major mechanism by which the β1 subunit and MRCKα alone are mediating its effects on improved lung function and repair.

The alveolar capillary barrier is comprised of two physical barriers: a tight alveolar epithelial barrier composed of cuboidal ATII cells and flattened ATI cells which account for the majority of the alveolar epithelial cell population, and a relatively more permeable microvascular endothelial barrier^7^. Microvascular filtration provides a fluid source to the lung and selectively limits the permeability to large protein molecules, like albumin, while the tight alveolar epithelial barrier contributes more than 90% of the resistance to albumin flux. As such, even a slight increase in epithelial permeability leads to a notable impact on lung fluid balance^39^. In healthy lungs, the alveolar capillary barrier system is important for maintaining pulmonary fluid homeostasis which is also reflected in the cardiogenic lung edema that contains much less protein compared with edema originating from ARDS. Such cardiogenic lung edema could be quickly resolved due to the relatively undamaged alveolar capillary barrier integrity since net AFC depends on intact barrier function and unimpaired fluid clearance capacity^8^. This link between barrier function and fluid clearance is further exemplified in studies with human lungs not used for transplant, in which levels of claudin-4, a major tight junction protein, directly correlated with alveolar fluid transport following ex vivo perfusion^40^. Further, it is well established that there is a strong correlation between impaired AFC capacity and mortality in ARDS patients^9^. While this is true, without intact epithelial and endothelial barriers, there can be no, or at least insufficient, net removal of fluid from a leaky lung. While improved barrier function in either epithelial, endothelial, or both cell layers would be beneficial, in the current study, it is still unclear whether gene transfer of MRCKα into lungs with existing injury restored ZO-1 and occludin levels in alveolar epithelial cells, microvascular endothelial cells, or both cell types. However, our studies in cultured ATI cells^23^ and in MVECs in the present study showing that MRCKα or β1 gene transfer can increase junctional complex protein levels, as well as our finding that the loss of VE-cadherin expression in lungs of LPS-injured mice is attenuated following gene transfer of MRCKα or β1, suggests that barrier function is being regulated in both cell types in vivo.

Previously, we found that gene transfer of β1-Na^+^, K^+^-ATPase into mouse lungs and cultured cells increased tight junction (ZO-1, occludin) protein abundance and plasma membrane localization^18^. In studying the mechanisms by which the β1 subunit of the Na^+^, K^+^-ATPase upregulates tight junction protein levels, we identified MRCKα as a β1 subunit-interacting protein through mass spectrometry^23^. Cell culture experiments in primary rat alveolar epithelial type I cells demonstrated that knockdown or inhibition of MRCKα abrogated β1 subunit-increased tight junction protein expression and TEER. Further, overexpression of MRCKα alone in the cultured cells significantly increased ZO-1 membrane localization and basal TEER, indicating that MRCKα is a downstream mediator of the β1 subunit, conveying β1’s signal to tight junction complex formation and barrier function; overexpression of MRCKα alone is sufficient to recapitulate the upregulation of epithelial barrier function by β1 in vitro^23^. Our results here show that gene delivery of MRCKα alone not only increased tight junction protein levels in healthy mice, but also restored ZO-1 and occludin expression and lung barrier function in pre-injured living lungs, indicating that downregulation of MRCKα might play a role in the pathogenesis of ARDS, and specifically, might be involved in the pulmonary barrier disruption. The fact that activation of MRCKα by either gene transfer or overexpression of the β1-Na^+^, K^+^-ATPase in lungs with existing injury is able to treat the disease provides a rationale for developing easier to administer pharmacological agents to activate MRCKα for ARDS.

MRCKα is a serine/threonine protein kinase and downstream effector of Cdc42, a small Rho GTPase that regulates actin-myosin cytoskeleton contraction in various cell physiological processes, including cell adhesion, polarity, morphology and motility^41,42^. In non-muscle eukaryotic cells, such as lung epithelial and endothelial cells, actin-myosin (non-muscle myosin II) crosslinking forms a belt-like perijunctional actomyosin ring (PJAR) structure that encircles cells at the cytoplasmic surface and regulates intercellular barrier function through interacting and stabilizing adhesion complexes, including tight junctions and adherens junctions^43,44^. Although MLC kinase (MLCK) is the major enzyme responsible for MLC phosphorylation, Cdc42-mediated MRCKα activation directly phosphorylates MLC at Ser19, inducing actin-myosin contraction and coupling to the plasma membrane^42,45^. In HeLa cells transfected with plasmid expressing Flag-tagged MRCKα, the strong staining signal along the cell periphery and cell–cell junction area also indicated that MRCKα might regulate activity of proteins involved in paracellular barrier function^46^. Indeed, in endothelial cells, it has been reported that MRCKα and MRCKβ are recruited to cell–cell junctions by Rap1 through the localization of CDC42 to the same cell–cell contacts to ultimately induce non-muscle myosin II and actin organization^26^. MRCKα also indirectly activates MLC through phosphorylation of MYPT1 and therefore, inhibition of MLC dephosphorylation^42^. In all these cases, MRCKα appears to regulate junctional complexes by inducing cascades of phosphorylation events of key enzymes that regulate actin dynamics to recruit tight junction and adherens junction proteins to cell–cell contacts, but MRCKα has not been shown previously to affect the levels of tight junction proteins themselves. However, it is well established that a number of tight junction and adherens junction components can regulate transcriptional activity themselves or through interactions with binding partners^47^. Thus, transcriptional and post-transcriptional regulation of junctional complex proteins by MRCKα or its downstream targets is not unprecedented and could be the result of any number of mechanisms. So far there is no genetic susceptibility of ARDS identified to link to MRCKα, although we did find that MRCKα expression was significantly decreased in the lungs of patients with ARDS compared with controls^23^. However, several genes that regulate actomyosin contraction, promote focal adhesion kinase formation, and contribute to the assembly of intercellular junction complexes, have been recognized to relate to ARDS susceptibility. For example, MLCK regulates endothelial and epithelial barrier permeability through direct phosphorylation of MLC and variation in MLCK expression has been associated with the susceptibility to ARDS^48,49^. Similarly, bioactive lipid sphingosine-1-phosphate (S1P)-mediated cellular events have been reported to induce MLC phosphorylation, activation of Rho GTPase, and the recruitment and assembly of adhesion junction molecules and genetic variants of the S1P receptor S1PR3 have been shown to be positively associated with the risk of ARDS^50^.

Experiments utilizing cultured alveolar epithelial type I cells have shown that transient overexpression of MRCKα significantly increases occludin and ZO-1 plasma membrane localization and TEER, indicating that overexpression of MRCKα alone is sufficient to recapitulate the same upregulated epithelial barrier function induced by overexpression of the β1-Na^+^, K^+^-ATPase. Moreover, the results here in mice with existing lung injury demonstrate that gene transfer of either β1 or MRCKα alone attenuated the LPS-induced lung injury to a similar degree, indicating that, as in cultured cells, overexpression of MRCKα has the comparable capacity to treat ALI/ARDS as does β1. Surprisingly, when the two genes were delivered to the lungs at the same time, co-delivery of MRCKα and β1-Na^+^, K^+^-ATPase failed to further enhance any measured outcome compared with that seen following gene transfer of either protein alone, indicating there is no additive or synergistic effect mediated by simultaneous overexpression of both genes. Since the expression levels of the two transgenes are similar when delivered either alone or in combination, these results suggest that a maximal therapeutic effect has been achieved by either single gene transfer alone. Indeed, in early studies evaluating electroporation for gene delivery in the rat lung, electroporation of β1-expressing plasmids increased β1 expression by twofold while delivery of a β1-expressing adenovirus gave almost 40-fold increased expression of β1^16^. However, both conditions gave the same degree of increased alveolar fluid clearance in isolated lungs, which was also indistinguishable from the maximal effect achieved by Na^+^, K^+^-ATPase stimulation by procaterol, suggesting that a maximal physiological response (in this case AFC) had been achieved even at low levels of transgene expression and could not be further enhanced^16^. In the present study, gene transfer of either β1-Na^+^, K^+^-ATPase or MRCKα alone probably reaches the maximal therapeutic effects for treating ARDS. Taken together, these results suggest that while β1 enhances both AFC and barrier function, the enhancement of barrier function alone achieved following MRCKα delivery is sufficient to provide maximal treatment effect for the injured lung.

## Materials and methods

### Plasmids

The plasmid pcDNA3 was obtained from Promega (Madison, WI, USA). Plasmid pCMV6- Na^+^, K^+^-ATPase β1 expressing Myc-DDK-tagged human Na^+^, K^+^-ATPase β1 subunit is from Origene (Rockville, MD, USA). Plasmid pY340- MRCKα, expressing Flag-tagged human MRCKα was a generous gift from Dr. Paolo Armando Gagliardi (University of Bern)^25^. Plasmids were purified using Qiagen Giga-prep kits (Qiagen, Chatsworth, CA, USA) and suspended in 10 mM Tris–HCl (pH 8.0), 1 mM ethylenediaminetetraacetic acid (EDTA) and 140 mM NaCl.

### Transfection and induction of LPS injury in microvascular endothelial cells

Human lung MVECs (Lonza, Walkersville, MD, USA) were cultured on 0.1% gelatin-coated coverslips in 12-well plates in endothelial growth medium 2 (EGM2) containing 10% FBS and bullet kit additives (BioWhittaker, Walkersville, MD, USA). Cells were transfected with plasmids (2 µg DNA/well) using Lipofectamine 3000 (Invitrogen, Carlsbad, CA, USA) and 48 h later, LPS (1 µg/ml, #L2280; Sigma, St. Louis, MO, USA) was added in EGM2 containing 3% FBS and no growth factors. Five hours later, cells were washed with PBS, fixed with 4% paraformaldehyde for 10 min, and immunofluorescence for VE-cadherin was carried out as described^51^.

### In vivo gene transfer and induction of acute lung injury (ALI)

Male C57BL/6 mice (8–10 weeks) were anesthetized with isoflurane (2–4%) and 100 μg of each plasmid were delivered in 50 μl of 10 mM Tris–HCl (pH 8.0), 1 mM EDTA and 140 mM NaCl to mouse lungs by oropharyngeal aspiration and electroporation with eight 10-ms square wave pulses at field strength of 200 V/cm using externally placed pediatric pacemaker electrodes (Medtronic, Redmond, WA, USA) on both sides of mouse chest with an ECM830 electroporator (BTX, Harvard Apparatus, Holliston, MA, USA)^52^. ALI was induced by Escherichia coli 055: B5 LPS (Sigma-Aldrich, St. Louis, MO, USA) as described previously^18^. Briefly, after anesthesia LPS was delivered by oropharyngeal aspiration at 5 mg/kg in 50 μl total volume for each mouse. Mice were challenged with LPS alone or 24 h before gene transfer (n = 6 mice/group). Mice were euthanized 48 h after gene transfer (3 days after LPS administration). All animal studies were approved by the University of Rochester Committee on Animal Resources and experimental procedures were carried out under the institutional guidelines for the care and use of laboratory animals in an American Association for the Accreditation of Laboratory Animal Care-approved facility. All animal studies complied with the ARRIVE guidelines.

### Western blot analysis

Western blot was performed as previously described^18^. Briefly, half of the left lobe of mouse lungs were homogenized in 300 μl of lysis buffer (1× Reporter lysis buffer (Promega, Madison, WI, USA), containing protease inhibitor (Roche, Basel, Switzerland)) using a TissueLyser II (Qiagen, Germantown, MD, USA). Supernatants were used for SDS-PAGE and western blots. Thirty micrograms of total protein were loaded on 10% SDS-PAGE gels, transferred onto PVDF membrane and probed with primary antibodies against DDK tag (Origene, Rockville, MD, USA), ZO-1, occludin, and VE-cadherin (Invitrogen, Rockford, IL, USA), β1-Na^+^, K^+^-ATPase (Millipore, Billerica, MA, USA), MRCKα (Santa Cruz, Dallas, TX, USA) and glyceraldehyde-3-phosphate dehydrogenase GAPDH (EMD Millipore, Burlington, MA, USA). Data were analyzed using the NIH Image J software.

### Measurement of wet to dry ratios

The wet to dry (W/D) ratio evaluates total pulmonary water content. It was measured 72 h after LPS instillation. After sacrificing mice, lungs were excised and weighed immediately for wet lung weight values. Stable dry lung weight values were obtained after lungs were placed in an oven at 70 °C for 72 h.

### BAL analysis

BAL analysis to measure total protein concentration and cellularity in BAL fluid was performed as described previously^17^. Briefly, 0.7 ml of sterile PBS was instilled into mouse lungs and lavaged twice. The BAL fluid was collected and separated for supernatant and cell pellet, respectively. The total protein concentration of the supernatant was measured using a Bradford assay (Bio-Rad), and BAL albumin concentration was measured with a mouse albumin ELISA quantitation kit (Bethyl Laboratories, Montgomery, TX, USA). The total number of cells from BAL was counted using a hemocytometer and cells then were stained with Diff-Quik (Siemens, Newark, DE, USA) after cytospin.

### Histological analysis

Histological analysis was to evaluate the inflammatory response and pathological changes in the lung. Immediately following euthanasia by Nembutal overdose, lungs were inflated to the pressure of 20 cm H_2_O with buffered formalin, removed, fixed overnight, and paraffin-embedded. Sections (5 µm) were stained with hematoxylin and eosin.

### Lung permeability assay

Pulmonary permeability was measured by the leakage of Evans Blue Dye (EBD) labeled albumin from blood into airways as described^18,53^. Briefly, EBD (30 mg/kg) was administrated by tail-vein injection 47 h after gene transfer. One hour later, lungs were perfused with sterile PBS to remove EBD in the vasculature and then excised and dried at 60 °C. One day later, EBD was extracted in formamide (Fisher Scientific, Pittsburgh, PA, USA) and quantified by absorbance at 620 and 740 nm, using the formula E_620_ (EBD) = E_620_ − (1.426 × E_740_ + 0.030)^54^.

### Measurement of AFC in live mice

AFC was evaluated by quantifying the rate of removal of an EBD labeled albumin isosmolar (324 mOsm) solution from the alveolar airspace^18,33^. AFC was calculated by the equation:

$${\text{AFC}} = 1 - \left( {{{{\text{C}}_{0} }/{{\text{C}}_{30} }}} \right),$$

where C_0_ is the initial concentration of the isosmolar solution before instillation and C_30_ is the protein concentration obtained after 30 min of mechanical ventilation. Mice were preoxygenated with 100% oxygen using a rodent ventilator (MiniVent Type 845, Harvard Apparatus, Holliston, MA, USA) for two minutes prior to instillation of EBD and the animals were maintained at 37° using a circulating water heating pad throughout the course of the measurement. Procaterol (a specific β2AR agonist, 10^−8^ mol/L) was administered in the instillate as a positive control.

### Statistical analysis

Each experiment was repeated at least three times with the number of animals per cohort indicated for each figure (n = 3–10). The data of each series is displayed as mean values ± standard error of the mean (SEM) unless otherwise noted. Graphing and statistical comparison of the data were performed using Prism 8 (GraphPad Software, San Diego, CA, USA). Measurements for more than two groups were analyzed by one-way ANOVA and multiple comparisons. P values less than 0.05 were considered to be statistically significant (Supplementary Information).

## Supplementary Information

Supplementary Information.
