# Supplementary material for: Gene transfer of MRCKα rescues lipopolysaccharide-induced acute lung injury by restoring alveolar capillary barrier function
Source: Sci Rep. 2021 Oct 21;11:20862. doi: 10.1038/s41598-021-99897-3 (PMC8531330; doi:10.1038/s41598-021-99897-3)

Supplemental Figure 1. Full Western Blot Images for Figure 1.

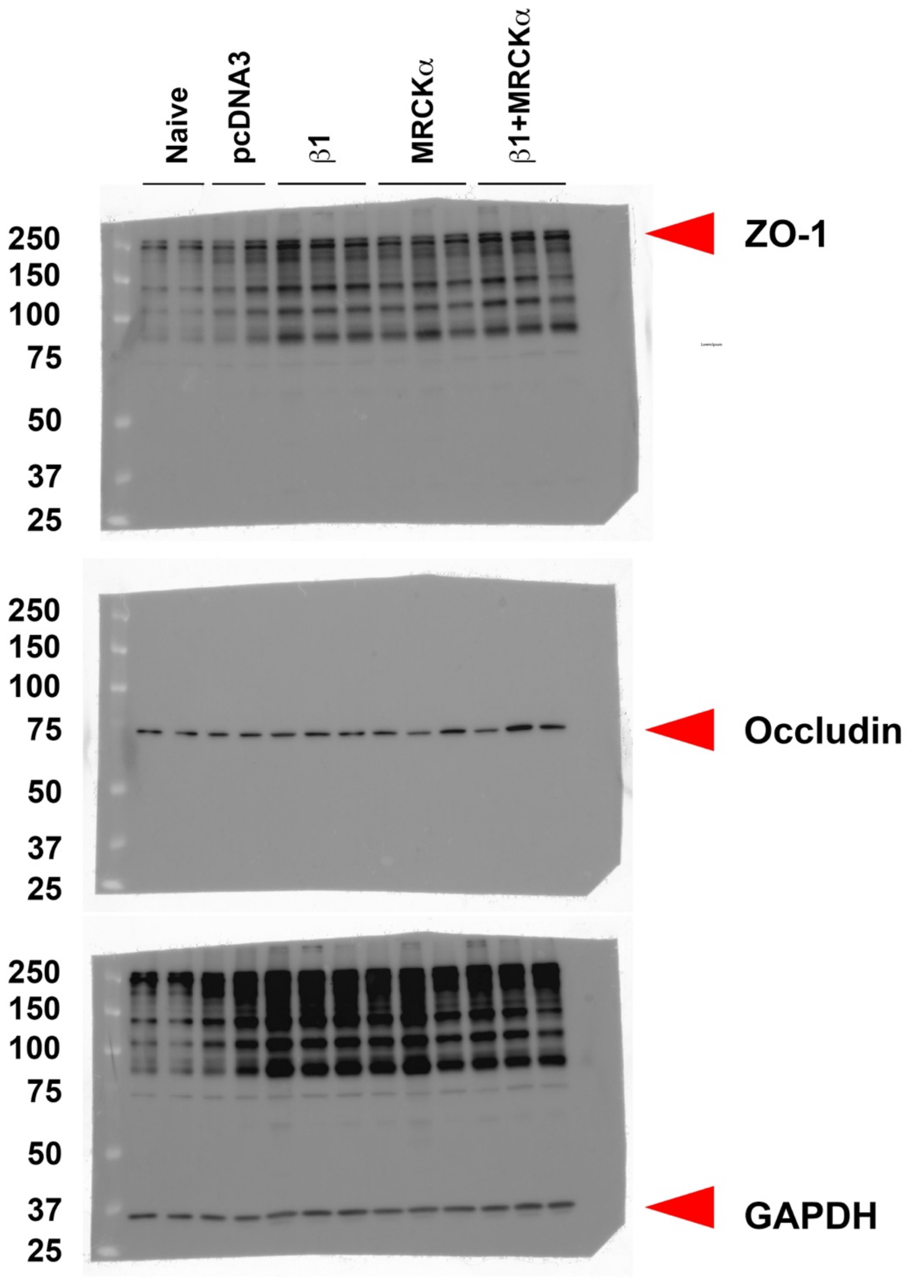

Supplemental Figure 2. Full Western Blots for Figure 3.

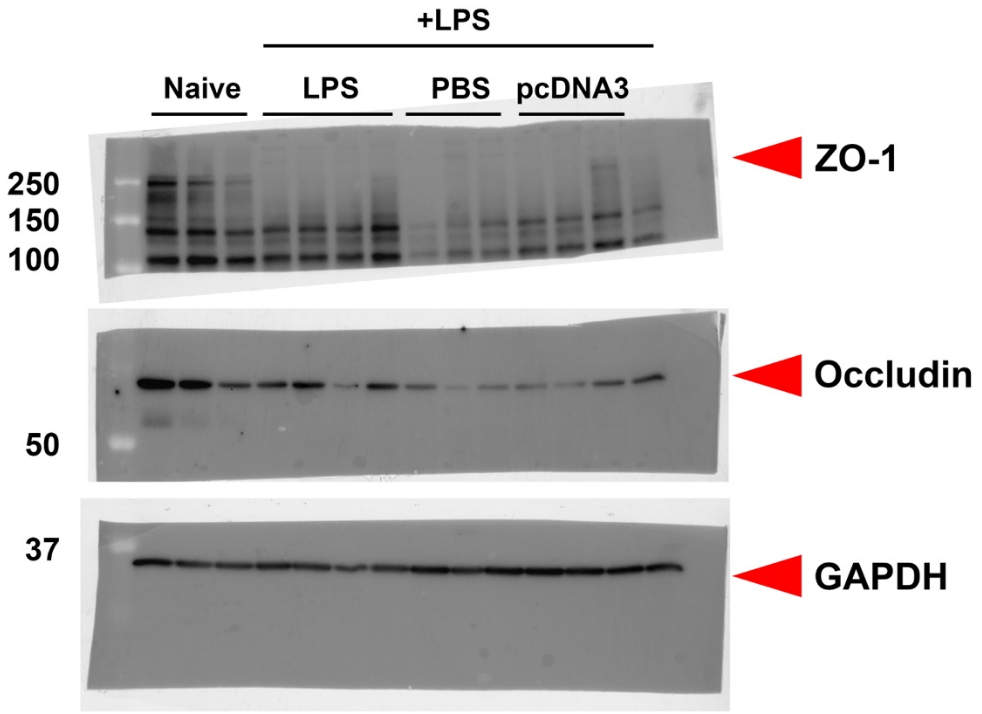

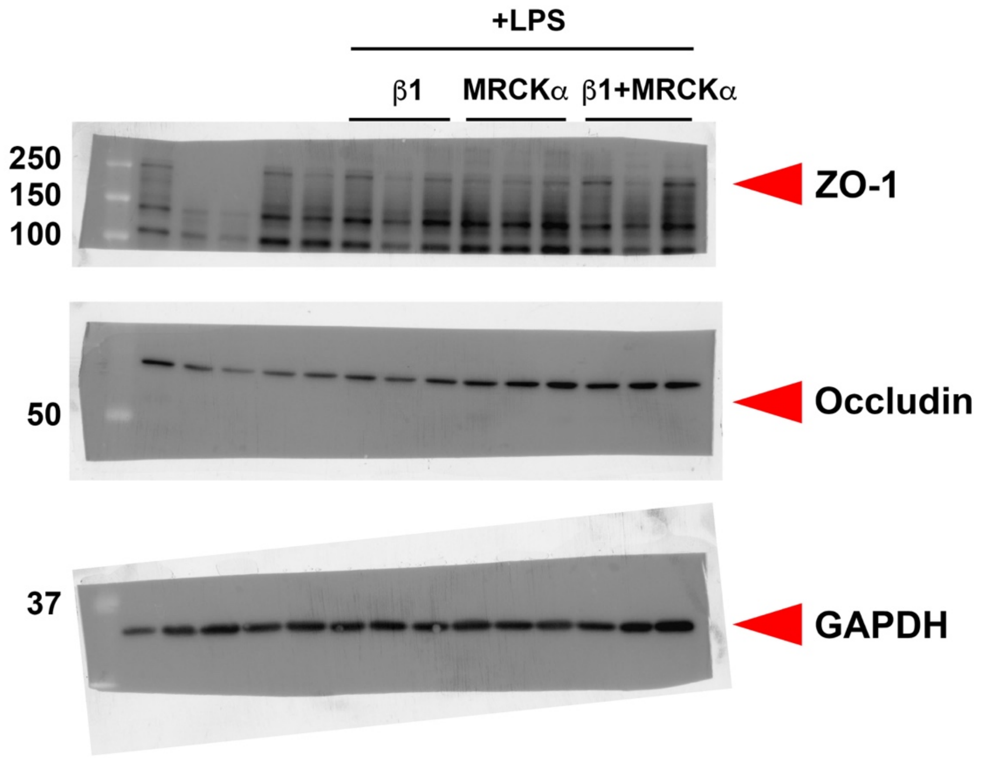

Supplemental Figure 3. Full Western Blots for Figure 5

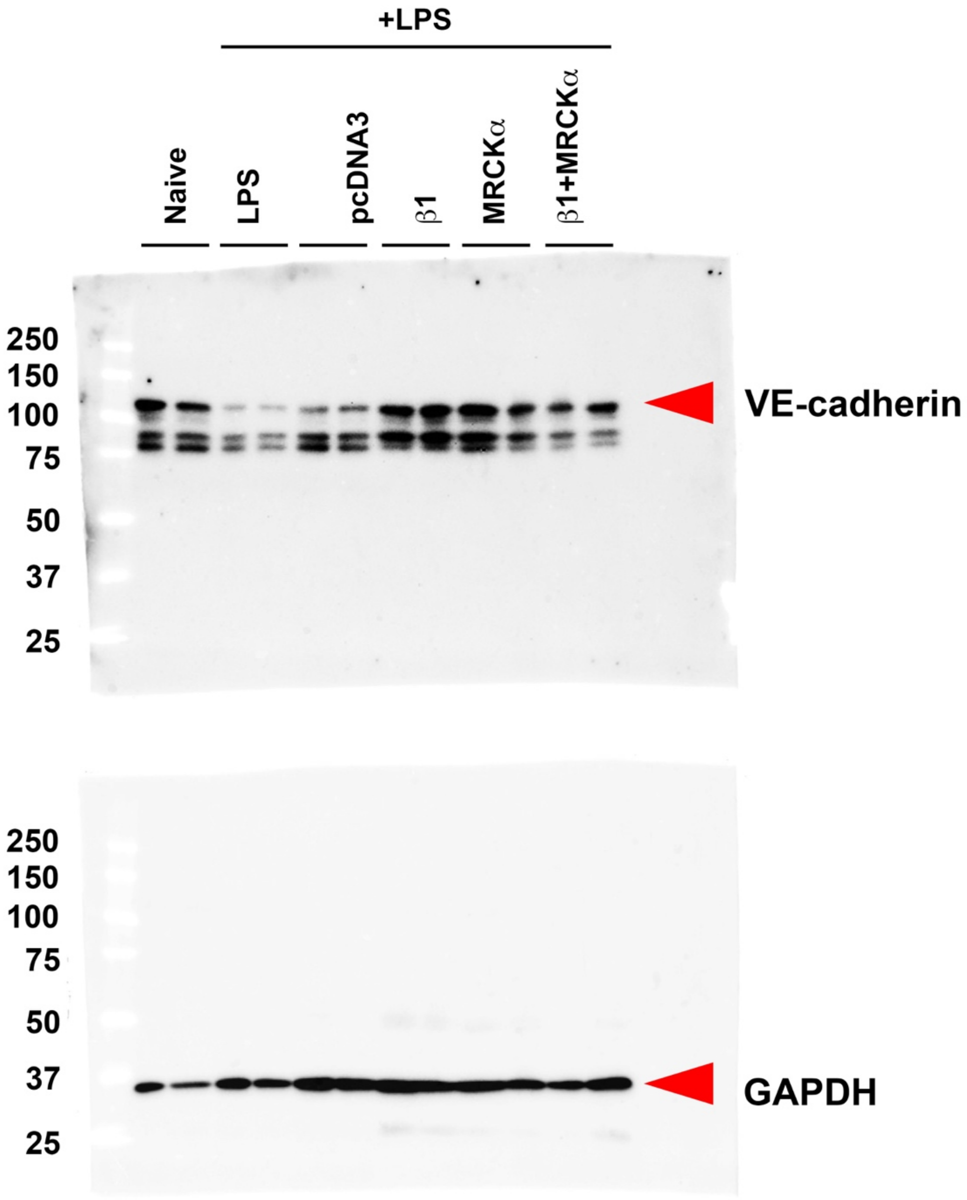

Supplement: Supplementary file 1 — Supplementary Information. [file 41598_2021_99897_MOESM1_ESM.pdf]
